# Specificity of Childhood Trauma Type and Attenuated Positive Symptoms in a Non-Clinical Sample

**DOI:** 10.3390/jcm8101537

**Published:** 2019-09-25

**Authors:** Arielle Ered, Lauren M. Ellman

**Affiliations:** Department of Psychology, Temple University, Philadelphia, PA 19122, USA; arielle.ered@temple.edu

**Keywords:** childhood trauma, attenuated positive symptoms, subthreshold psychosis, multiple victimization

## Abstract

Background: Childhood traumatic experiences have been consistently associated with psychosis risk; however, the specificity of childhood trauma type to interview-based attenuated positive psychotic symptoms has not been adequately explored. Further, previous studies examining specificity of trauma to specific positive symptoms have not accounted for co-occurring trauma types, despite evidence of multiple victimization. Methods: We examined the relationship between childhood trauma (Childhood Trauma Questionnaire) with type of attenuated positive symptom, as measured by the Structured Interview for Psychosis-risk Syndromes (SIPS) among a non-clinical, young adult sample (*n* = 130). Linear regressions were conducted to predict each attenuated positive symptom, with all trauma types entered into the model to control for co-occurring traumas. Results: Results indicated that childhood sexual abuse was significantly associated with disorganized communication and childhood emotional neglect was significantly associated with increased suspiciousness/persecutory ideas, above and beyond the effect of other co-occurring traumas. These relationships were significant even after removing individuals at clinical high-risk (CHR) for psychosis (*n* = 14). Conclusions: Our results suggest that there are differential influences of trauma type on specific positive symptom domains, even in a non-clinical sample. Our results also confirm the importance of controlling for co-occurring trauma types, as results differ when not controlling for multiple traumas.

## 1. Introduction

One-third of the general population will experience traumatic life events (TLEs) at some point in their lifetime [1]. Further, TLEs occurring in childhood are associated with numerous negative outcomes, spanning from chronic health problems to various types of psychopathology [1,2], and psychosis spectrum disorders in particular [3,4]. Traumatic life events are a robust environmental predictor of psychotic symptom severity and spontaneous relapse in individuals with schizophrenia [3,5]. Childhood trauma has also been found to have a dose-dependent relationship with psychosis, with the more traumatic events experienced predicting greater amounts of psychotic symptoms in those with schizophrenia [6], and exposure to multiple types of traumas, also termed multiple or poly-victimization, has been associated with even greater risk for developing psychotic symptoms [7,8]. Further, studies of individuals at clinical high-risk (CHR) for psychosis reveal that trauma usually occurs prior to the onset of CHR symptoms [9], implying a causal relationship.

More recently, some studies have moved to a model of examining psychosis along a continuum, particularly in non-clinical samples of individuals with psychotic-like experiences (PLEs), or subthreshold, subclinical attenuated versions of positive symptoms, and individuals experiencing attenuated positive symptoms (APS), or more distressing, clinically-relevant subthreshold psychotic symptoms, indicating CHR status [10]. Individuals with PLEs and APS share genetic, phenomenological, and environmental risk factors with individuals with frank psychosis, including a history of TLEs [10,11,12]. Incidences of trauma have also been found to be significantly associated with PLEs in our prior work [13,14,15,16]. Examining individuals with PLEs or APS rather than those diagnosed with schizophrenia spectrum disorders or at CHR for psychosis helps to minimize the contributions of potential confounds, such as illness-related factors that may impact symptoms and outcomes, and antipsychotic medication usage [17,18].

When examining the relationship between specific types of early-life adversities to specific psychotic or attenuated positive symptoms, research has primarily focused on help-seeking samples of individuals with schizophrenia spectrum disorders and individuals at CHR. In examinations of individuals with chronic schizophrenia, childhood sexual abuse has been associated with hallucinations, even after controlling for paranoia [19,20]. Examinations of records from community mental health centers have also found that patients reporting hallucinations (particularly auditory and visual hallucinations) and delusions had experienced significantly more childhood adversities than those without these symptoms [6], and physical and sexual abuse in particular have been associated with hallucinations in individuals with schizophrenia [21]. However, despite some evidence of specificity between symptom and trauma type within individuals in the chronic course of schizophrenia, meta-analytic examinations reveal that all trauma subtypes are associated with psychotic symptoms [3], and some studies have argued that examining individual TLE types rather than all TLEs combined might in fact obscure the effect of trauma on psychosis, which may account for inconsistent findings in prior studies [22].

Within CHR individuals, total number of traumatic events has been significantly associated with perceptual disturbances and interpersonal traumas have been associated with suspiciousness [9]. Other CHR samples have demonstrated that both violent (including physical and sexual abuse) and non-violent traumas predicted elevated grandiosity, and that violent traumas predicted heightened suspiciousness in low psychosis-risk psychiatric controls [23]. Additional CHR samples have found that physical abuse was specifically related to suspiciousness and disorganization, and both childhood sexual abuse and emotional abuse were related to grandiosity [24]. Further, rates of perceptual abnormalities, suspiciousness, and grandiosity were found to be higher in CHR individuals with a history of physical abuse than those without a history of trauma [25]. This evidence from the CHR literature paints a mixed picture, as it appears that many types of traumas predict to specific APS with little agreement between studies.

Previous work in non-clinical samples has linked childhood abuse to an overall subthreshold positive symptom domain and subthreshold paranoia [26]; however, this work combined incidences of physical and emotional neglect into a neglect item and physical, emotional, and sexual abuse into an abuse item. A large study examining hallucinations in the general population found that childhood neglect and childhood sexual abuse were related to visual hallucinations, and childhood sexual abuse and physical abuse were related to tactile hallucinations [27]; however, this study examined frank hallucinations within a non-clinical sample rather than APS or PLEs and did not assess for other positive symptoms, such as delusions. Other studies within the same sample revealed that neglect was associated with paranoia [28]; however, the neglect construct consisted of a single question: “You were severely neglected as a child”. Another study examined victimization in females in the United Kingdom and found that lifetime sexual trauma predicted hallucinations, and multiple incidences of victimization predicted thought interference/control, paranoia, strange experiences, and hallucinations [29]. Notably, the study’s measurement of PLEs utilized a questionnaire-based measure which included only one item for each type of PLE (e.g., thought interference/control, strange experiences). Further, Saha and colleagues [30] examined relationships between TLEs and subthreshold delusions in a community sample and found that lifetime TLE exposure increased the risk for delusional-like experiences two-fold, and that this relationship was dose-dependent, with each added trauma type predicting more delusional-like experiences. However, this study assessed for delusional-like experiences only and did not assess for other types of PLEs, including hallucinatory experiences, grandiosity, or disorganized communication. Finally, another study [31] utilized a large population-based sample and found significant relationships between childhood abuse (physical or sexual) and both paranoia and verbal hallucinations, but utilized questionnaire-based assessments of PLEs and did not assess for other APS such as grandiosity or disorganization.

Most notably, no paper attempted to control for the presence of co-occurring traumas in non-clinical populations, despite considerable evidence of the effect of multiple victimization [7,8]. In studies examining victimization of children in a non-clinical sample, 71% reported experiencing one trauma, and 69% of those individuals who experienced victimization had experienced one or more additional types of trauma within the year [32]. Given the evidence of multiple victimizations, studies of the psychosis spectrum should account for co-occurrence of trauma types when attempting to predict from trauma type to specific attenuated positive symptoms.

Previous work across phase of illness indicates that results are mixed when predicting specific types of positive psychotic symptoms or APS from specific traumas. Thus, the current study sought to better examine the specificity of childhood trauma type to each attenuated positive symptom in a sample of non-clinical individuals by an interview-based symptom assessment, controlling for the effect of other co-occurring traumas. We hypothesized that, once controlling for co-occurring traumas, we would not find specificity between trauma type and attenuated positive symptom type.

## 2. Materials and Method

### 2.1. Participants

Study participants included 130 undergraduate students at a large, racially/ethnically and socioeconomically diverse urban university, who were recruited through the university’s online psychology subject pool. Participants received course credit for completing questionnaires and were compensated monetarily for clinical interviews. Participant demographics are presented in Table 1. The study was approved by Temple University’s Institutional Review Board (Approval Number 13359). All participants provided informed consent prior to participation.

### 2.2. Procedure

Participants completed questionnaires on study visit 1. Individuals who endorsed 8 or more distressing positive psychotic-like symptoms on the Prodromal Questionnaire (PQ; see measures) were classified as high PLE (*n* = 99) and individuals endorsing 3 or fewer distressing PLEs (the mean from our pilot data) and no more than 8 total PQ positive items were classified as low PLE (*n* = 31). These participants were invited back for study visit 2, which included clinical interviewing for APS using the Structural Interview for Psychosis-risk Syndromes (SIPS; see Measures). The two groups did not differ on any demographic variables, but individuals in the high PLE group did have significantly more experiences of emotional abuse and physical and emotional neglect (Table 1).

### 2.3. Measures

The Prodromal Questionnaire’s (PQ; [33]) 45-item positive subscale was used as the questionnaire-based measure of psychotic-like experiences (PLEs). Endorsing 8 or more distressing PLEs has been validated against the SIPS with 90% sensitivity and 49% specificity [33]. This measure was used to select individuals with high (8 or more distressing PLEs endorsed) and low (3 or fewer distressing PLEs endorsed) PLEs to return for interview-based assessment of APS.

The Structured Interview for Psychosis-risk Syndromes (SIPS; [34]) was used to determine the presence of attenuated positive psychotic symptoms, as well as to determine presence of psychosis-risk syndromes. The SIPS is the most commonly used interview for assessing psychosis-risk syndromes within the United States [35] and has well-validated interrater reliability and predictive validity of conversion to psychosis [36,37]. Scores on a SIPS item of 0–2 represent subthreshold psychotic symptoms, score of 3–5 represent clinically relevant symptoms that could indicate psychosis-risk, and a score of 6 represents frank psychotic symptoms. Participants across the range of SIPS scores were retained. The five attenuated positive symptoms from the SIPS were used (Unusual Thought Content/Delusional Ideas, Suspiciousness/Persecutory Ideas, Grandiosity, Perceptual Abnormalities/Hallucinations, Disorganized Communication).

The Childhood Trauma Questionnaire Short Form (CTQ; [38]) was used to assess traumatic life events occurring before the age of 16. This inventory, validated for ages 12 and older, assesses five types of childhood maltreatment (Emotional Abuse, Physical Abuse, Sexual Abuse, Emotional Neglect, and Physical Neglect). The CTQ shows good sensitivity and specificity, internal consistency, and convergent validity in both clinical and community samples [39]. The sum score of each trauma subtype was used.

### 2.4. Data Analysis

First, given the post-hoc nature of these analyses, a power analysis was conducted. The dependent variables (SIPS Positive Items 1–5) were then examined for normality visually and statistically. Next, bivariate Pearson’s correlations were examined between all study variables. Linear regressions were conducted for each of the five SIPS attenuated positive symptom items (Unusual Thought Content/Delusional Ideas, Suspiciousness/Persecutory Ideas, Grandiosity, Perceptual Abnormalities/Hallucinations, and Disorganized Communication) using all CTQ subscales (Emotional Abuse, Physical Abuse, Sexual Abuse, Emotional Neglect, and Physical Neglect) simultaneously as independent variables, in order to determine if a specific trauma subtype was related to the attenuated positive symptom above and beyond the all other co-occurring trauma subtypes. Finally, individuals at clinical high-risk for psychosis (CHR, *n* = 14) were removed and models were re-run to determine if these individuals are driving results, or if results are associated with the transdiagnostic phenotype of attenuated positive symptoms.

## 3. Results

A power analysis indicated that we were powered at the .95 level to detect medium effect sizes (Cohen’s *f*^2^ = 0.12). As SIPS data were skewed (kurtosis = −0.020–9.897, skewness = 0.889–3.137); a constant of one was added and a log transformation was applied to each item to achieve normality. Bivariate correlations revealed significant associations between all trauma subtypes (*r* = 0.214–0.618, all *p*’s < 0.05; see Table 2 for full correlation matrix of all study variables), indicating that traumas do co-occur within our sample. Thus, all types of trauma were entered into the model to determine the specific effect of each trauma type on each attenuated positive symptom tested, controlling for all other types of trauma.

Linear regressions revealed that emotional neglect was significantly associated with increased suspiciousness/persecutory ideas (standardized β = 0.34, *t* (128) = 2.95, *p* = 0.004), controlling for all other trauma types. Additionally, childhood sexual abuse was significantly associated with increased disorganized communication (standardized β = 0.19, *t* (128) = 1.98, *p* = 0.05), controlling for other traumas. There were no significant relationships between traumas and interview-based unusual thought content/delusional ideas, grandiosity or perceptual abnormalities/hallucinations (see Table 3 for values). After removing individuals at CHR and rerunning the models, the relationship between sexual abuse and disorganization was still significant (standardized β = 0.12, *t* (115) = 1.13, *p* = 0.01), as was the relationship between emotional neglect and suspiciousness/persecutory ideas (standardized β = 0.27, *t* (115) = 2.09, *p* = 0.04) (See Table 4). After applying a Bonferroni correction for multiple comparisons, a *p* value of 0.01 would be considered significant. Thus, the relationship between suspiciousness/persecutory ideas remains significant while the childhood sexual abuse—disorganized communication relationship is no longer significant, but rather trended toward significance.

## 4. Discussion

This is the first study to our knowledge to demonstrate the relationship between specific attenuated positive symptoms and specific types of trauma in a non-clinical sample utilizing interview-based assessments of APS and controlling for poly-victimization. Contrary to our hypotheses, we found that emotional neglect in childhood was associated with paranoid ideation/suspiciousness and childhood sexual abuse was associated with disorganization, both above and beyond the influences of co-occurring traumas. Further, we found no significant relationships between trauma types and assessments of grandiosity, perceptual abnormalities, or unusual thought content, after controlling for co-occurring traumas. Our findings were consistent after removing individuals at CHR for psychosis, suggesting that the associations between traumas and specific attenuated positive symptoms could occur in the course of a range of disorders and are not specific to psychosis-risk. In particular, attenuated positive symptoms occurs in many disorders including mood disorders, trauma-related disorders, and borderline personality disorder [40,41]; therefore, our findings are likely more pertinent to a cross-diagnostic phenotype rather than CHR for psychosis specifically.

Our unique results may be attributable to the non-clinical nature of the population combined with the interview-based assessment of five domains of attenuated positive symptoms and the consideration of poly-victimization, of which this is the first study to report on such results. These findings point to sexual abuse and emotional neglect as predicting to disorganization and paranoid ideation respectively when interview-based assessments of non-clinical individuals are examined, and thus these results are uniquely important when considering a transdiagnostic phenotype of APS. This is emphasized by our findings remaining significant even after removing individuals at CHR (see Table 4), which lends further credence to TLEs relating to APS themselves and particularly disorganization, which occurs across diagnostic categories, rather than psychotic disorders specifically.

Given the differences between results utilizing bivariate correlations which do not control for co-occurring traumas (see Table 2), and regression models that control for co-occurring trauma types (see Table 3), multiple victimization clearly plays a role within this sample. By not controlling for multiple victimization, results may overestimate relationships between particular trauma types and APS.

The current study has several strengths, including a non-clinical sample, which increases generalizability to the general population. However, the sample was acquired from an undergraduate population, which may limit generalizability. Even so, the university from which it was obtained is quite large and diverse racially, ethnically, and socioeconomically, which may mitigate some generalizability concerns. There were some additional limitations of this study which future studies may choose to address. This study utilized a cross-sectional design, and thus we cannot infer temporal order of the variables; however, the questions pertaining to trauma were about childhood (presumably before the study time point). We also included a retrospective measure of childhood trauma. While the CTQ is a well-validated measure of childhood trauma, recent studies point to suboptimal agreement between retrospective and prospective measurements of childhood maltreatment, noting that 52% of individuals who reported childhood maltreatment prospectively did not report this maltreatment retrospectively [42], and underreporting of TLEs in retrospective assessment has been confirmed by meta-analytic examinations as well [3]. The use of retrospective measures of childhood maltreatment, such as the CTQ, may in fact underestimate the prevalence of trauma within the sample. Further, the CTQ does not provide information on the timing and sequence of traumatic events. The current study also was limited by selecting participants based on high and low PLE status (with overrepresentation of high PLEs in this subsample), which may not be generalizable to the full continuum of PLEs, especially at mid and lower levels of the continuum. Future studies should employ prospective longitudinal designs and utilize biological data (e.g., genetics) to better capture accurate reports of childhood trauma, as well as to better elucidate pathways from childhood trauma to attenuated positive symptoms and eventual frank psychotic symptoms. Further, future studies should also examine the role of other variables that may influence the trauma–APS relationship, such as substance or medication usage and demographic factors such as race and perceived discrimination.

## 5. Conclusions

These findings resolve several issues prominent within the psychosis and trauma symptom specificity literature. This is the first study in a non-clinical population to account for multiple victimization when exploring the relationship between trauma type and specific attenuated positive symptoms. By controlling for co-occurring trauma types, we are able to better elucidate the underlying relationships between trauma type and APS in a more naturalistic way, as traumas often co-occur in the real world. Finally, it is notable that the APS related to trauma in our results (paranoid ideation and disorganization) occur across many disorders, such as anxiety [40,41,43,44], mood [40,41,45,46], personality [47,48], trauma-related [40], and obsessive-compulsive and related disorders [41,49,50], and are not specific to schizophrenia spectrum disorders alone. Thus, these results have broad implications for the general population and risk for psychopathology. Through early identification and treatment, the effect of traumas occurring in childhood (such as sexual abuse and emotional neglect) may be ameliorated before the development of more chronic psychopathologies.

## Figures and Tables

**Table 1:** Demographics and clinical features of sample.

	Total Sample (*n* = 130)	High PLE (*n* = 99)	Low PLE (*n* = 31)	Difference (*t* or *χ*^2^, *p*)
Age (years) mean (SD)	19.68 (1.9)	19.74 (2.09)	19.48 (1.12)	−0.61, 52
Gender % (*n*) Male	20% (26)	21% (21)	16% (5)	0.38, 0.54
Ethnicity % (*n*) Hispanic	0.8% (1)	1	0	0.32, 0.57
Race % (*n*)	-	-	-	4.36, 0.36
Asian	15% (19)	16% (16)	10% (3)	-
African American	13% (17)	11% (11)	19% (6)	-
Caucasian	59% (77)	60% (59)	58% (18)	-
Multiracial	8% (10)	9% (9)	3% (1)	-
Unknown	5% (7)	4% (4)	10% (3)	-
Clinical High Risk Status % (*n*)	11% (14)	14% (14)	0% (0)	-
Emotional Abuse M (SD)	10.35 (5.09)	11.32 (5.11)	7.26 (3.62)	4.11, <0.001 *
None to Low	46% (60)	36% (36)	77% (24)	16.51, 0.001 *
Low to Moderate	22% (28)	24% (24)	13% (4)	
Moderate to Severe	13% (17)	16% (16)	3% (1)	
Severe to Extreme	19% (25)	23% (23)	6% (2)	
Physical Abuse Total M (SD)	7.15 (3.28)	7.38 (3.40)	6.39 (2.75)	1.48, 0.14
None to Low	69% (90)	65% (64)	84% (26)	4.25, 0.236
Low to Moderate	15% (20)	17% (17)	10% (3)	
Moderate to Severe	7% (9)	8% (8)	3% (1)	
Severe to Extreme	9% (11)	10% (10)	3% (1)	
Sexual Abuse Total M (SD)	6.83 (4.36)	7.15 (4.78)	5.81 (2.37)	1.51, 0.14
None to Low	75% (97)	73% (73)	77% (24)	4.21, 0.240
Low to Moderate	7% (9)	5% (5)	13% (4)	
Moderate to Severe	8% (11)	9% (9)	6% (2)	
Severe to Extreme	10% (13)	12% (12)	3% (1)	
Emotional Neglect Total M (SD)	10.08 (4.7)	10.86 (4.76)	7.58 (3.52)	3.54, 0.001 *
None to Low	53% (69)	46% (46)	74% (23)	8.442, 0.038 *
Low to Moderate	28% (37)	31% (31)	19% (6)	
Moderate to Severe	11% (14)	12% (12)	6% (2)	
Severe to Extreme	8% (10)	10% (10)	0% (0)	
Physical Neglect Total M (SD)	6.88 (2.6)	7.19 (2.74)	5.90 (1.72)	2.47, 0.02 *
None to Low	69% (90)	65% (64)	84% (26)	5.833, 0.120
Low to Moderate	16% (21)	19% (19)	6% (2)	
Moderate to Severe	9% (12)	9% (9)	10% (3)	
Severe to Extreme	6% (7)	7% (7)	0% (0)	
CTQ Total Score M (SD)	41.29 (14.97)	43.91 (12.59)	32.94 (8.63)	3.74, <0.001 *
SIPS ^1^ Unusual Thought Content M (SD) (range = 0–4)	90 (1.24)	1.06 (1.31)	0.39 (0.80)	2.71, 0.01 *
SIPS Paranoia/Delusional Ideas M (SD) (range = 0–5)	1.15 (1.26)	1.38 (1.27)	0.39 (0.92)	4.05, <0.001 *
SIPS Grandiosity M (SD) (range = 0–4)	0.28 (0.78)	0.36 (0.87)	0.03 (0.18)	2.09, 0.04 *
SIPS Perceptual Abnormalities M (SD) (range = 0–5)	0.71 (1.13)	0.85 (1.22)	0.26 (0.63)	2.59, 0.01 *
SIPS Disorganized Communication M (SD) (range = 0–4)	0.60 (0.87)	0.70 (0.91)	0.29 (0.64)	2.31, 0.02 *
PQ ^2^ Positive Total Score M (SD) (range = 0–38)	16.84 (9.53)	20.84 (6.92)	4.06 (3.60)	12.94, <0.001 *
Anxiety Disorder Diagnosis % (*n*)	55% (72)	62% (61)	36% (11)	2.60, 0.01 *
Depressive Disorder Diagnosis % (*n*)	46% (60)	53% (52)	26% (8)	2.65, 0.009 *
Bipolar Disorder Diagnosis % (*n*)	5% (7)	7% (7)	0% (0)	1.52, 0.13
PTSD ^3^ Diagnosis % (*n*)	13% (17)	16% (16)	3% (1)	1.88, 0.06
OCD ^4^ Disorder Diagnosis % (*n*)	10% (13)	10% (10)	10% (3)	0.07, 0.95
Alcohol Use Disorder Diagnosis % (*n*)	27% (35)	27% (27)	26% (8)	0.16, 0.87
Substance Use Disorder Diagnosis % (*n*)	24% (31)	25% (25)	19% (6)	0.67, 0.51

* = significant difference between groups; ^1^ = Structured Interview for Psychosis-risk Syndromes; ^2^ = Prodromal Questionnaire; ^3^ = Posttraumatic Stress Disorder; ^4^ Obsessive-Compulsive Disorder.

**Table 2:** Correlations of all attenuated symptom variables and all trauma variables (*n* = 130).

	Unusual Thought Content (UTC)	Paranoid Ideation	Grandiosity	Perceptual Disturbances	Disorganization	PA	EA	SA	EN	PN
SIPS ^1^ UTC	-	0.580 **	0.260 **	0.408 **	0.277 **	−0.069	0.003	−0.032	0.118	0.070
SIPS Paranoia		-	0.261 **	0.356 **	0.404 **	0.116	0.213 *	0.048	0.315 **	0.107
SIPS Grandiosity			-	0.169	0.213 *	0.000	−0.006	−0.030	−0.035	−0.005
SIPS Perceptual Disturbances				-	0.410 **	0.030	0.128	0.006	0.211 *	0.191 *
SIPS Disorganization					-	0.194 *	0.273 **	0.283 **	0.266 **	0.248 **
Physical Abuse (PA)						-	0.586 **	0.353 **	0.341 **	0.311 **
Emotional Abuse (EA)							-	0.482 **	0.618 **	0.444 **
Sexual Abuse (SA)								-	0.337 **	0.214 *
Emotional Neglect (EN)									-	0.528 **
Physical Neglect (PN)										-

* *p* < 0.05; ** *p* < 0.001; ^1^ Structured Interview for Psychosis-risk Syndromes.

**Table 3:** Regressions for attenuated positive symptoms and all trauma types (*n* = 130). Bonferroni corrected significant *p*-value = 0.01.

	Unstandardized β	SE	Standardized β	*p*
**Model 1: Unusual Thought Content**				
Emotional Abuse	−0.005	0.016	−0.040	0.771
Physical Abuse	−0.018	0.019	−0.100	0.364
Sexual Abuse	−0.006	0.013	−0.043	0.670
Emotional Neglect	0.021	0.015	0.171	0.160
Physical Neglect	0.008	0.024	0.038	0.722
**Model 2: Paranoia/Delusional Ideas/Suspiciousness**				
Emotional Abuse	0.009	0.015	0.079	0.549
Physical Abuse	0.003	0.019	0.015	0.891
Sexual Abuse	−0.012	0.013	−0.091	0.353
Emotional Neglect	0.042	0.014	0.342	0.004 **
Physical Neglect	−0.021	0.023	−0.094	0.357
**Model 3: Grandiosity**				
Emotional Abuse	0.003	0.010	0.034	0.809
Physical Abuse	0.001	0.013	0.006	0.956
Sexual Abuse	−0.003	0.009	−0.033	0.746
Emotional Neglect	−0.004	0.010	−0.054	0.661
Physical Neglect	0.002	0.016	0.014	0.898
**Model 4: Perceptual Abnormalities/Disturbances**				
Emotional Abuse	0.005	0.014	0.045	0.741
Physical Abuse	−0.010	0.018	−0.063	0.562
Sexual Abuse	−0.009	0.012	−0.076	0.451
Emotional Neglect	0.019	0.013	0.169	0.160
Physical Neglect	0.024	0.021	0.118	0.263
**Model 5: Disorganized Communication/Disorganization**				
Emotional Abuse	0.005	0.012	0.051	0.695
Physical Abuse	0.004	0.015	0.026	0.806
Sexual Abuse	0.020	0.010	0.191	0.050 *
Emotional Neglect	0.009	0.011	0.094	0.413
Physical Neglect	0.023	0.018	0.127	0.209

** *p* < 0.01; * *p* ≤ 0.05.

**Table 4:** Regressions for attenuated positive symptoms and all trauma types without individuals at CHR (*n* = 116). Bonferroni corrected significant *p*-value = 0.01.

	Unstandardized β	SE	Standardized β	*p*
**Model 1: Unusual Thought Content**				
Emotional Abuse	−0.001	0.016	−0.010	0.951
Physical Abuse	−0.014	0.019	−0.088	0.452
Sexual Abuse	0.000	0.013	0.002	0.989
Emotional Neglect	0.012	0.016	0.108	0.432
Physical Neglect	0.013	0.023	0.062	0.575
**Model 2: Paranoia/Delusional Ideas/Suspiciousness**				
Emotional Abuse	0.012	0.016	0.110	0.465
Physical Abuse	0.009	0.019	0.054	0.631
Sexual Abuse	−0.005	0.013	−0.045	0.673
Emotional Neglect	0.033	0.016	0.273	0.039 *
Physical Neglect	−0.024	0.022	−0.112	0.292
**Model 3: Grandiosity**				
Emotional Abuse	0.007	0.010	0.106	0.502
Physical Abuse	0.007	0.012	0.066	0.572
Sexual Abuse	−0.002	0.008	−0.025	0.822
Emotional Neglect	−0.011	0.010	−0.152	0.268
Physical Neglect	0.005	0.015	0.037	0.738
**Model 4: Perceptual Abnormalities/Disturbances**				
Emotional Abuse	0.014	0.014	0.154	0.323
Physical Abuse	−0.013	0.016	−0.092	0.425
Sexual Abuse	0.001	0.011	0.006	0.955
Emotional Neglect	0.001	0.014	0.010	0.939
Physical Neglect	0.021	0.020	0.117	0.287
**Model 5: Disorganized Communication/Disorganization**				
Emotional Abuse	0.011	0.012	0.135	0.356
Physical Abuse	−0.002	0.015	−0.012	0.910
Sexual Abuse	0.026	0.010	0.266	0.011 *
Emotional Neglect	−0.001	0.012	−0.014	0.913
Physical Neglect	0.020	0.018	0.116	0.261

* *p* ≤ 0.05.
